# Effects on Brood Development in the Carpenter Ant *Camponotus vicinus* Mayr after Exposure to the Yeast Associate *Schwanniomyces* *polymorphus* Kloecker

**DOI:** 10.3390/insects12060520

**Published:** 2021-06-04

**Authors:** Mark E. Mankowski, Jeffrey J. Morrell, Patricia K. Lebow

**Affiliations:** 1 Forest Products Laboratory Starkville, USDA Forest Service, Starkville, MS 39759, USA; 2 Centre Timber Durability and Design Life, University of the Sunshine Coast, Sippy Downs, QLD 4102, Australia; jmorrell@usc.edu.au; 3 Forest Products Laboratory Madison, USDA Forest Service, Madison, WI 53726, USA; patricia.k.lebow@usda.gov

**Keywords:** artificial diet, infrabuccal pocket, *Camponotus vicinus*, B vitamins, ectosymbiont, defaunation, sterols, yeast exposure, *Schwanniomyces polymorphus*

## Abstract

**Simple Summary:**

Carpenter ants are important to ecosystem services as they assist in the breakdown of course woody debris when excavating wood for nests. Feeding on a variety of carbohydrate and protein sources, they have an infrabuccal filter that limits passage of large food particles to their gut. A variety of yeasts have been found associated with the infrabuccal pocket and the nests of these ants. The yeast *Schwanniomyces polymorphus* is associated with the carpenter ant *Camponotus vicinus*. To examine a possible nutritional association between this yeast and ant, we reared small sub-colonies of defaunated and non-defaunated *C. vincus* brood on several artificial diets where various nutritional components were removed. Part of the testing involved exposure of brood to these diets and cells of *S. polymorphus*. Dietary treatments that were augmented with yeast generally had deleterious effects on brood development compared to diets without yeast. However, increased brood weight and increased number of adult ants from initial brood was observed in non-defaunated ants fed a diet where B vitamins and sterols were absent, but augmented with live yeast. The results suggest a supplemental role for this yeast in ant nutrition and development.

**Abstract:**

The yeast *Schwanniomyces polymorphus* is associated with the infrabuccal pocket in the carpenter ant *Camponotus vicinus* (Hymenoptera: Formicidae), but its role in ant development is poorly defined. The potential effects of this yeast on brood development were examined on sets of larval groups and workers over a 12 week period. Worker–larval sets were fed variations of a completely artificial, holidic diet and exposed or not exposed to live *S. polymorphus.* Worker–larval sets in half of the experiment were defaunated using a two-step heat and chemical process. Brood development and number of adult ants produced were significantly affected by the heat/chemical defaunation process. Compared to worker–larval groups fed a basal, complete diet, all treatments resulted in no or deleterious larval development. Brood weights and number of worker ants produced from the original larval sets at initiation were significantly higher in non-defaunated ant groups fed a diet lacking both B vitamins and cholesterol and exposed to live *S. polymorphus*. We propose that this yeast may help ants to more efficiently assimilate nutrients when fed nutrient-deficient diets, particularly those deficient in sterols.

## 1. Introduction

Carpenter ants (*Camponotus* spp. Hymenoptera: Formicidae) belong to a large ecologically successful genus of over 1000 species [1,2]. Although carpenter ants generally nest in wood, they do not use it as a food source. In some Nearctic regions, a few species can be structural nuisance pests [2,3]. However, the majority of *Camponotus* species play an important role in Holarctic forest ecosystems where they aid in the breakdown of woody debris through their nesting habits and feed omnivorously on plant exudates and live arthropod prey including pest insects. Carpenter ants do not ingest solid food particles as workers possess a buccal filter or infrabuccal pocket that limits the passage of large food particles to the gut [4,5,6,7,8]. Incoming food filtered by the infrabuccal pocket forms a pellet that is squeezed by muscular action, and any liquid produced passes to the midgut before the pellet is evacuated. Previous studies observed Gram-negative bacteria and yeast species in the infrabuccal pocket and galleries of *Camponotus vicinus* Mayr; however, the potential function, if any, of these organisms has not been examined [8,9,10].

Many studies examining the relationship of carpenter ants with associated microorganisms have focused on midgut symbionts, but the biota and potential associates of the infrabuccal pocket remain unknown [11,12,13,14,15]. In the US Pacific Northwest, the yeast *Schwanniomyces* *polymorphus* Kloecker is associated with the carpenter ant *Camponotus vicinus*, whereby it is the predominate yeast in the galleries and buccal cavities of these ants [10]. Studies examining other ants have identified *Schwanniomyces* spp. (formerly *Debaryomyces* spp.) associated with the *Formica rufa* L., *Formica aquilonia* Yarrow, and *Formica polyctena* Foerst [16,17,18,19,20]. The relationship between *Formica* and this yeast remains unknown, but it was highlighted by these studies since the yeasts were found only in active ant colonies. Yeasts associated with nests and ant larvae of the fire ant, *Solenopsis invicta* Buren, produced sterols required for larval development [21,22,23]. This is important from a nutritional standpoint as most insects cannot generate sterol compounds [24,25,26]. 

Yeasts have been found associated with leaf cutting ants (*Atta* spp.) where they appear to be involved in plant biomass metabolism in the ants’ fungal gardens [27,28,29,30]. The European paper wasp, *Polistes dominula* Christ, has been shown to supply a specialized ecological niche for the yeast *Saccharomyces cerevisiae* Hansen in its alimentary tract [31,32]. Larval pupation in the stingless bee, *Scaptotrigona depilis* Moure, was shown to require ingestion of the yeast *Zygosaccharomyces* spp. to obtain the necessary steroid precursors from sterols produced by the fungus to produce molting hormones [33].

Endosymbiont yeasts are also present in many beetle species where they are passed into eggs prior to or during oviposition [18,34,35]. Synthesis of required nutrients absent from diets such as B complex vitamins or sterols by microorganisms is common in many insects [24,25,36,37,38]. Synthesis of B vitamins, particularly thiamine, nicotinic acid, and biotin, as well as sterols, on minimal media by yeast species is well documented [24,25,35,39]. 

Studies examining the metabolic effects of vitamins and sterols in insects often use complete artificial (holidic) diets containing known quantities of specific nutrients. Components are deleted, and the effects on insect growth are compared to a complete or basal diet [24,40,41,42,43]. Studies examining experimental diets on ant larvae can be complicated since the ant larvae are kept in groups where individual larvae are indistinguishable and, therefore, it is difficult to discern the effects of a diet on individuals [44,45,46,47,48]. In studies where the nutritional effects of potential ectosymbiotic or endosymbiont microorganisms are involved, the host insects are often rendered aposymbiotic via defaunation (without microorganisms). Defaunation can be achieved via chemical and supra-optimal temperatures or a combination of both [14,34,49,50]. Early studies on the nutritional requirements of the carpenter ant *Camponotus pennsylvanicus* De Geer showed limited brood development when dried Brewer’s yeast was removed from an artificial diet fed to this species [51,52]. The nutritional association of *Camponotus vicinus* with its yeast associates has not been examined. The consistent association of the yeast *S. polymorphus* with *C. vicinus* galleries and the infrabuccal cavity [10] coupled with previous reports of yeast/ant interactions suggest a possible nutritional role for this yeast. In two other studies, we examined the effects of heat/chemical defaunation, as well as B vitamin and sterol deletion, on small colonies of *C. vicinus* [50,53], and we found that diets deficient in sterols had deleterious effects on ant brood development when compared to ants fed a complete (basal) diet. The objective of the current study was to examine the potential nutritional role of live yeast on ant brood development and production of adult worker ants where groups of non-defaunated and defaunated ants and larvae were fed diets lacking various components and exposed to this yeast associate.

## 2. Materials and Methods 

### 2.1. Artificial Diet Preparation 

The compositions of the diets tested were based on previous studies [40,41,43,53] and were prepared according to [53] with the exception that a smaller concentration of riboflavin was used in this study as we found development to be better using the lower amount [53]. The artificial diet contained fatty acids, fat-soluble vitamins, amino acids, inorganic salts, water-soluble vitamins, and sucrose. All dietary components were purchased from Fisher Scientific. We developed this complete or basal diet so that individual components could be deleted. The complete (all nutrient containing) chemically defined holidic diet without deletions is referred to as the basal diet throughout this paper and in the figures. The components and preparation of this diet were prepared as described below (Table 1). 

Fatty acids and fat-soluble vitamins were weighed in 5 mL beakers and then dissolved with 2 mL of 60 °C ethanol. This was poured into 80 mL of 60 °C deionized water to which 0.5 mL of Tween-80 was added. This solution was adjusted to 100 mL with 60 °C sterile deionized water. Forty milliliters of this solution was used per 100 mL of diet, and the amounts of fatty acids were adjusted accordingly.

An amino-acid portion was prepared by adding dry amino acids to a 250 mL flask and dissolving with 40 mL of the fatty-acid solution along with 9 mL (one ml of each) of mineral salt from the previously prepared stock solutions and 6 mL of a ribonucleic acid (RNA) solution. The pH was adjusted to 6.5 with 1.5 mL of 1 M KOH. This mixture was stirred for 10 min, autoclaved at 120 °C for 15 min, and then allowed to cool. 

The RNA solution that was added was prepared by adding 2 mL of 1 M KOH to 3 mL of hot water and then pouring this solution over the ribonucleic acid to dissolve it. Any remaining RNA solution was removed by a 1 mL rinse with warm sterile water.

The water-soluble portion of the diet was prepared by placing 10 g of sucrose in a 50 mL beaker and adding 1 mL of each vitamin from stock solutions to dissolve the sucrose. Vitamins were added in micrograms per gram of diet according to [41,53]. After the sucrose was completely dissolved, the mixture was placed in a syringe, sterile deionized water was added to bring the mixture to 42.0 mL, and the pH was adjusted to 6.5 by adding 1.5 mL of 2 N K_2_PO_4_. The resulting mixture was filtered using a 0.22 μm filter into the non-water-soluble portion described above. Sterile deionized water was added in place of a given stock solution aliquot when a component was deleted. This prepared liquid basal diet and solutions with components removed were refrigerated at 5 °C until needed.

### 2.2. Effects of Diets Supplemented with Schwanniomyces polymorphus 

The effects of adding live *Schwanniomyces polymorphus* to diet treatments with all or some nutritional components removed was assessed by comparing groups of ants fed the basal (complete) diet and diets deficient in various nutritional components to those fed the same diets but supplemented with live yeast cultures. We also examined the effect of a pre-feeding test defaunation treatment on one half of the study. Ants were collected near Corvallis, Oregon from two wild colonies of *C. vicinus* during winter diapause and stored at 5 °C for 2 weeks prior to test initiation.

A total of 16 treatments were tested, each consisting of eight replicates of eight workers with 15 third to fourth instar larvae. Each worker colony was a mimic of a small satellite colony in that it was queenless. Ants from the two different colonies were not mixed together in the replicates. Each replicate of workers and brood was placed in a separate petri dish with small holes on the top to provide air to the workers and brood. A small piece of sponge was placed in each petri dish and periodically wetted with 100 mL of sterile water to maintain adequate moisture conditions. 

One half of the experiment (a total of eight different diets) was exposed to a heat/chemical defaunation step and the other half was not. This resulted in the 16 treatments described below. To defaunate ant workers and brood, replicates were exposed to 39 °C for 48 h [34] and then fed a mixture of propiconazole-tetracycline to kill microbial symbionts [14,50]. Tetracycline was used to eliminate endosymbionts in the ant midguts [14]. The heat step was performed for the 48 h period one time before test initiation. The propiconazole–tetracycline mixture was fed to ants twice per week with regular test diet feedings for the first 4 weeks of the test. We did not, however, examine if this would eliminate the midgut endosymbionts completely, as this study was focused on the yeast associate. Propiconazole was used to inhibit yeast growth [50]. In an earlier study, we noticed that yeasts and fungi grew after feeding ants various concentrations of the propiconazole and tetracycline mixture [50]. We added heat treatment in combination with the chemical treatment according to [34], who found that supra-optimal temperatures stopped the growth of yeast symbionts in the beetle *Sitophilus oryzae* L. We tested elevated temperatures on *Schwanniomyces polymorphus* and found that this species did not grow at 39 °C [50]. The high-temperature exposure also appeared to control the growth of a parasitic Phorid fly, sometimes found in *C. vicinus* colonies [50]. After the 48 h heat exposure, the defaunation replicates were fed 100 μL of a 6.66 × 10^−4^ mg/mL propiconazole–tetracycline (equal parts) mixture in 0.5 M sucrose solution two times per week for the first 4 weeks of the test. In an earlier study [50], similarly treated ants were dissected after 1 week, with infrabuccal pocket contents plated on acid media and incubated for 5 days at 25 °C. Plates were then examined for yeast growth, and none was noted, indicating that yeasts were removed from the ants [50]. The heat and chemical treatment did not appear to have an effect on worker ant or larval mortality, as all workers and larvae survived the 4 day defaunation period before the test. No earlier studies have assessed yeast removal from larvae; thus, the effects of the pretreatment on any yeast in larvae were unknown.

The potential role of live *S. polymorphus* in ant nutrition was assessed by feeding each group of four replicates from each colony one of four different diets with or without exposure to live yeast cultures, which was repeated with another set of replicates exposed to the heat and chemical treatment prior to being fed the diets (4 replicates × 2 colonies × 4 diets × 2 exposures (yeast/no yeast) × 2 defaunation/no defaunation (heat/chemical or no heat chemical = 128 replicates). The use of a holidic basal diet enabled the deletion of various elements from the basal diet to examine their effects on brood development [53]. The sixteen treatments were as follows:

1. Basal diet (fed basal, complete diet only),
2. Basal diet + yeast (fed basal, complete diet + *S. polymorphus*),
3. No B vitamins (fed basal diet minus all B vitamins),
4. No B vitamins + yeast (fed basal diet minus all B vitamins + *S. polymorphus*),
5. No B vitamins, no cholesterol (fed basal diet minus all B vitamins and cholesterol),
6. No B vitamins, no cholesterol + yeast (fed basal diet minus all B vitamins and cholesterol + *S. polymorphus*),
7. Sugar water (fed sugar water only (0.5 M sucrose)),
8. Sugar water + yeast (fed sugar water only + *S. polymorphus*),
9. Heat/chemical basal diet (defaunation then fed basal diet),
10. Heat/chemical basal diet + yeast (defaunation then fed basal diet + *S. polymorphus*),
11. Heat/chemical, no B vitamins (defaunation then fed basal diet minus all B vitamins),
12. Heat/chemical, no B vitamins + yeast (defaunation then fed basal diet minus B vitamins + *S. polymorphus*),
13. Heat/chemical, no B vitamins, no cholesterol (defaunation then fed basal minus B vitamins, minus cholesterol),
14. Heat/chemical, no B vitamins, no cholesterol + yeast (defaunation then fed basal minus B vitamins, minus cholesterol + *S. polymorphus*),
15. Heat/chemical, sugar water (defaunation then fed sugar water only (0.5 M sucrose)),
16. Heat/Chemical, sugar water + yeast (defaunation then fed sugar water only + *S. polymorphus*).

A suspension of *S. polymorphus* that was isolated and identified in an earlier study using the BIOLOG^®^ microbial identification system (Biolog Inc., Hayward, CA, USA) [10] was grown in 125 mL of a previously described medium [39] containing no B vitamins or growth factors. Cultures were incubated on a rotary shaker at room temperature (22–23 °C) for 5 days and then passed through 0.22 μm filter paper using a Buchner funnel, 150 mL Erlenmeyer flask, and vacuum. The filtrate was discarded, and the yeast cells were washed off the filter paper with sterile distilled water and suspended in 20 mL of sterile water. One hundred microliters of yeast suspension was then applied directly on the groups of worker ants and larvae so that the workers would ingest the yeasts as they groomed themselves and the larvae. The yeast solution was applied two times per week over the 12 week test period. 

The worker groups in each replicate were fed their specific diet for 12 weeks at 3 day intervals by placing 100 μL of the diet on a small aluminum foil square cup [43,50,51,53,54]. Diets were replaced at 3 day intervals to avoid spoilage of the diet solution. All feedings were performed in a laminar flow hood to further minimize the risk of microbial contamination.

The replicate Petri dishes of ants and brood were kept in lidless clear plastic boxes at room temperature (22–23 °C) on a laboratory bench where they were exposed to natural light and monitored after each 3 day feeding interval. The role of the dietary treatments on ant development was assessed at 2 week intervals by removing developing brood with a fine horse-hair brush so they could be counted, weighed, and returned to their respective Petri dish. Adult worker ants produced in each Petri dish were counted at the end of the 12 week test period.

### 2.3. Yeast Isolations

At the end of the 12 week period, 20 worker ants were removed from each treatment and dissected. The infrabuccal pocket contents were removed and placed in 190 μL of sterile phosphate-buffered solution (PBS). Fifty microliters of this mixture was plated on acid medium and incubated at 25 °C for 5 days during which time the plates were examined for any signs of yeast growth. 

### 2.4. Statistical Analysis

Statistical tests for treatment differences are based on the 12 week exposure data; however, tables and profile plots of the unadjusted means and standard errors are also given to show trends over the duration of the experiment. Brood weight at the end of the test was fit as a four-factor linear model with heterogeneous variance structure based on heat by yeast groupings. In addition to the fixed factors of diet, yeast, and defaunation, colony was considered a fixed factor since the yeast and diet were specifically chosen with these particular colonies in mind; all interactions between the fixed factors were included in the model. According to the non-homogeneity exhibited in residual plots, initial GLMs were tested for homogeneity of variance with Levene’s test, which was rejected in each case. The final heterogeneous variance model was determined on the basis of reduced AIC (corrected Akaike’s information criteria) following Littell et al. [55]; moreover, homogeneity of the heat by yeast variances was tested. The number of adult worker ants produced from the original groups of 15 larvae were fit with a Poisson generalized linear model with replicates fit as a four-factor linear model. As with brood weights, diet, yeast, and defaunation, as well as colony, were considered fixed factors. However, because of computational issues and according to a pre-analysis with log-transformed values, no interactions involving colony were included; otherwise, all interactions among diet, yeast, and defaunation were included. Overdispersion was not significant but was considered borderline; hence, unit-level variation was modeled, and approximate F-tests were reported following Stroup et al. [56]. 

In addition to testing for interactions between the factors as noted above, final brood weights and the number of adult worker ants produced were compared to the basal diet, as well as between each specific diet treatment and that same diet with exposure to live yeast. Contrasts of least-square means were constructed to test for these differences in diets with significance at α = 0.05. Statistical analyses were conducted in SAS/STAT^®^ V14.1 software (proc mixed, and proc glimmix), SAS Version 9.4 software, SAS Institute Inc., Cary, NC, USA, 2016 [57]. 

## 3. Results

### 3.1. Effects of Diets on Ants Exposed to Schwanniomyces polymorphus

#### Brood Development and End Weight

**Figure 1:**
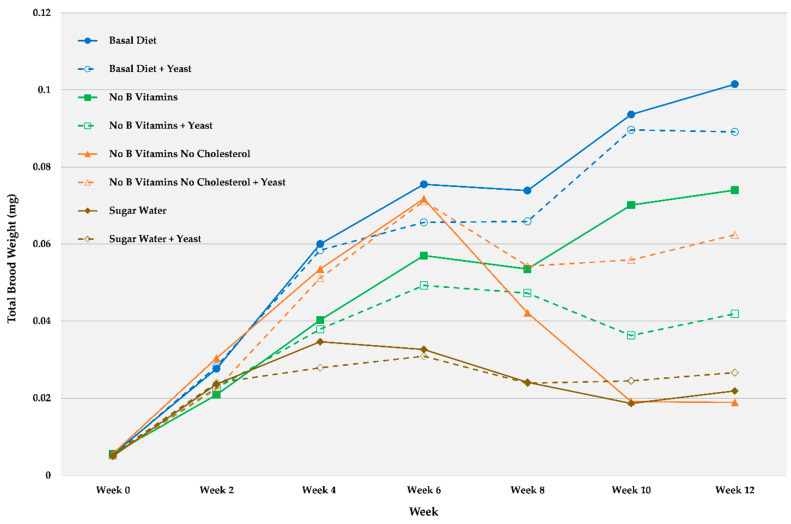
Average (*n* = 8) brood weights (mg) over 12 weeks for small worker colonies of *C. vicinus* (non-defaunated) fed various chemically defined diets with some nutrients deleted and the same diets augmented with a yeast associate.

Figure 1 and Table 2 show trends (nonstatistical) for average starting weights (time 0) and brood development at 2 week intervals over 12 weeks for non-defaunated ant and larval groups fed the various diets with and without yeast exposure. Brood weights remained highest for ants fed the basal (complete) diet. Table 2 was added to show standard errors for the means at each time point as they made the chart trends difficult to read. There was a notable increase in brood weight on all diets for the first 4–6 weeks, followed by either slight increases or decreases after 8 weeks. This appeared noticeable in diets lacking both B vitamins and cholesterol after 4 weeks (orange lines). Live yeast-exposed ants fed this same diet had higher brood weights (dashed orange line), suggesting that the yeast conferred some nutritional advantage to a diet lacking both B vitamins and cholesterol. This treatment and the sugar water only plus yeast resulted in increased brood weights. However, in the sugar water only diet plus yeast, the increase was very slight. The other yeast-augmented diets tended to result in lower brood weights compared to the diet without yeast, suggesting that yeast addition had a deleterious effect on development. 

**Figure 2:**
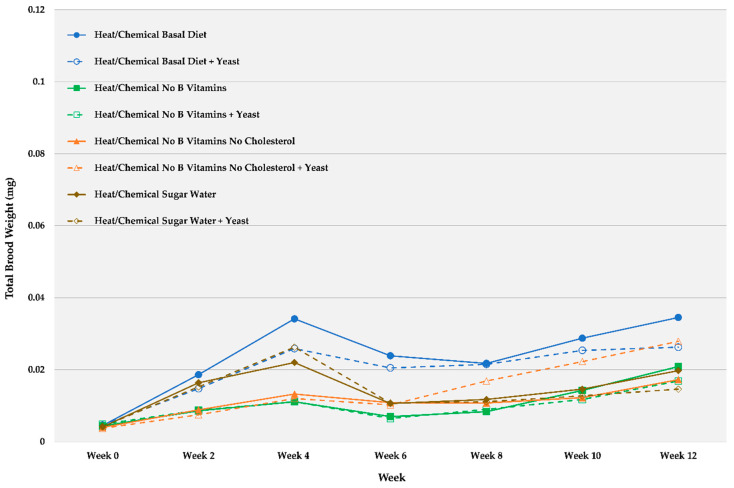
Average (*n* = 8) brood weights over 12 weeks for small worker colonies of *C. vicinus* defaunated using a two-step heat and chemical process and then fed chemically defined diets with some nutrients deleted and the same diets augmented with a yeast associate.

Figure 2 and Table 3 show trends (nonstatistical) for average starting weights (time 0) and brood development at 2 week intervals over 12 weeks for defaunated ant and larval groups fed the various diets with and without yeast exposure. Compared to the non-defaunated ant fed the same diet, the heat/chemical exposure to defaunate the ant and larval groups appeared deleterious to brood development. Brood weights remained highest for ants fed the basal (complete) diet. Table 3 was added to show standard errors for the means at each time point. There was an increase in brood weight on all diets for the first 4 weeks, followed by slight declines and then either a leveling out or slight increases after 8 weeks. Live yeast-exposed ant and larval groups where all B vitamins and cholesterol were removed from the diet were the only treatment to slightly gain weight compared to the same diet with no yeast starting at 6 weeks (orange dotted line), indicating that yeast exposure may have aided development.

**Figure 3:**
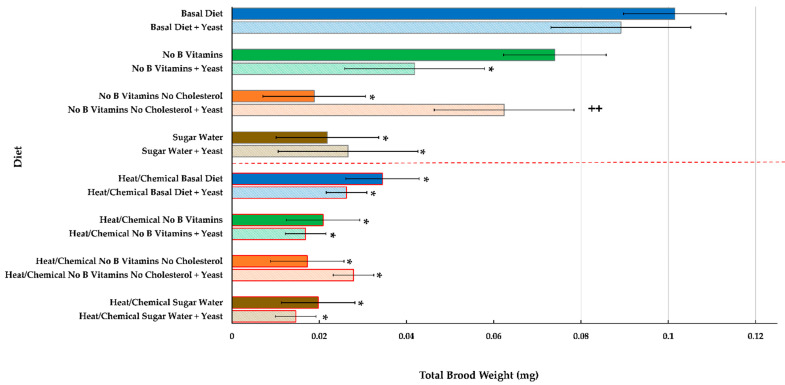
Total average (*n* = 8) brood weights at the end of 12 weeks for groups of workers and larvae of the ant *C. vicinus* either not exposed or exposed to a heat/chemical defaunation (red dotted line and red border bars) and fed eight different diets where various components were deleted and/or augmented with exposure to live yeast (*S. polymorphus*). Comparisons were made for all diets to the basal (all component) diet non-defaunated treatment (top blue bar), as well as with regard to yeast exposure or no yeast exposure. An asterisk (*) indicates *p* < 0.05 compared to the basal diet; double plus (++) indicates *p* < 0.05 compared to the same diet with or without yeast.

Figure 3 shows comparisons for the total average brood weight at the end of the 12 week period for all diets tested. Since the basal (all component) diet is considered the optimal diet for ant development according to nutritional value and the results of an earlier study using it [53], we designated it the control diet to compare the other diets. Even though brood developed better on this diet compared to other diets, it must be stated that artificial, holidic diets for ants are difficult to produce. A significant interaction between yeast exposure and diet was found (F_3,63_ = 3.18, *p* = 0.0298), indicating strong evidence that diet and yeast exposure affected brood weight, but in a nonadditive way. In addition, there was a significant defaunation before diet interaction (F_3,63_ = 5.33, *p* = 0.0024)). No interactions with colonies were detected (all *p* ≥ 0.0798), but an additive effect due to colony was detected (F_1,63_ = 15.70, *p* = 0.0002). Total average brood weights at the end of the 12 week test period were highest for non-defaunated larvae fed the basal diet compared to all other diets (Figure 3, top blue bar). All diets except the basal diet plus *S. polymorphus*, the diet lacking B vitamins, and the diet lacking B vitamins and cholesterol but with *S. polymorphus* resulted in significantly lower brood weights (Figure 3) compared to the basal diet. Brood weights were significantly higher (*p* = 0.0340) in groups exposed to a diet lacking both B vitamins and cholesterol but exposed to live yeast compared to the same diet without *S. polymorphus* (Figure 3, orange bars). The lower part of Figure 3 (red line and red outlined bars) shows that the heat/chemical defaunation treatment resulted in much lower brood weights at the end of the test for all diets tested. Defaunated ants and larvae exposed to yeast ended up with lower brood weights at the end of the test for all diets tested except the diet lacking both B vitamins and cholesterol. In this case, there was a slight increase in brood weight for ants fed this diet and exposed to *S. polymorphus*, but the increase was not significant as in non-defaunated ants exposed to yeast and fed this diet. It should be noted that the test for common variances across the defaunation by yeast groups was rejected (χ^2^ = 33.61, df = 3, *p* < 0.0001). 

### 3.2. Yeast Isolations 

Yeasts and other fungi were present in infrabuccal pocket contents of workers from all treatments at the end of the test period. *S. polymorphus* was isolated from workers in all treatments. Other yeast species and mold fungi were also isolated from these treatments, including a yeast that appeared to be a *Candida* spp. [39]. Plated *S. ploymorphus* and other yeasts were identified using BIOLOG^®^ software and via visual inspection [10]. The presence of other organisms was not surprising since no attempt was made to keep the treatments or ants sterile throughout the experiment.

#### Adult Number

**Figure 4:**
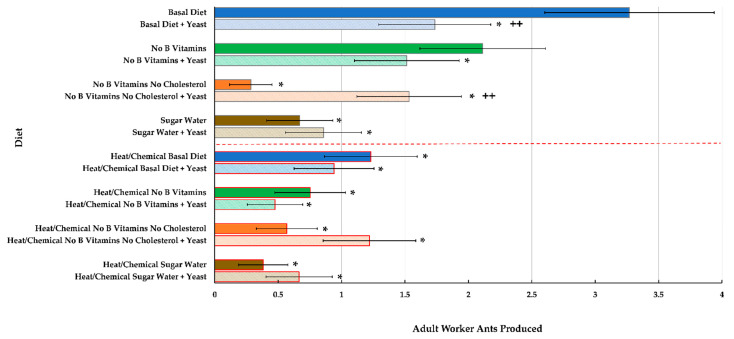
Estimated mean number of adult worker ants produced at the end of 12 weeks for groups of workers and larvae of the ant *C. vicinus* either not exposed or exposed to a heat/chemical defaunation (red line and red border bars) and fed eight different diets where various components were deleted and/or augmented with exposure to live yeast (*S. polymorphus*). Comparisons were made for all diets to the basal (all component) diet non-defaunated treatment (top blue bar), as well as with regard to yeast exposure or no yeast exposure. An asterisk (*) indicates *p* < 0.05 compared to the basal diet; double plus (++) indicates *p* < 0.05 compared between same diet with and without yeast.

There was also a significant interaction between yeast exposure and diet for number of adult ants reared from the original larval brood at the start of the test (F_3,111_ = 4.73, *p* = 0.0038). There was no significant defaunation before diet interaction on adult number (F_3,111_ = 2.15, *p* = 0.0767), and other defaunation interactions were also not significant (*p* ≥ 0.5910). All diets produced significantly fewer ants compared to the basal diet (Figure 4, top blue bar) except the diet minus B vitamins without yeast. In this case, however, there were still fewer adults than the basal diet, but the difference was not significant. As with brood weight, there were significantly more worker ants produced for the diet lacking both B vitamins and cholesterol but exposed to *S. polymorphous* (*t*_111_ = −2.64, *p* = 0.0096), suggesting that exposure to live yeast helped development on this limited diet (Figure 4, orange bars no red border). The lower part of Figure 4 (red line and red outlined bars) shows that the heat/chemical defaunation treatment resulted in much lower adult worker numbers at the end of the test for all diets tested (F_1,111_ = 8.17, *p* = 0.0051). Similar to end brood weights (Figure 3), defaunated groups of ants and larvae exposed to yeast ended up with lower brood weights at the end of the test for all diets tested except the diet lacking both B vitamins and cholesterol. In this case, there was a slight increase in brood weight for ants fed this diet and exposed to *S. polymorphus*, but the increase was not significantly different from non-defaunated ants exposed to yeast and fed this diet. The two colonies did produce a differing number of adult ants (F_1,111_ = 61.46, *p* = 0.0001), and their effect was modeled as a multiplicative factor across all the diets (via an additive term in the linear predictor for the link function of the Poisson rate parameter). The goodness-of-fit test for a Poisson model without modeling overdispersion was not significant (χ^2^ = 129.73, df = 111, *p* = 0.1081) but did indicate that a model with unit-level variation may be more appropriate. Our unit-level mixed Poisson model did estimate a nonzero unit-level variation and reduced the χ^2^/df to 0.90.

## 4. Discussion

Exposure to live *Schwanniomyces polymorphus* showed a positive effect on brood weights and number of worker ants produced in only one of the diet treatments exposed to yeast. The diet lacking both B vitamins and cholesterol plus yeast performed better than the same diet with no yeast exposure. Interestingly, brood weights from replicates exposed to a diet lacking all B vitamins but containing cholesterol and exposed to live yeast were not affected. This suggests that the yeast may have supplied sterol compounds that aided in brood development in the treatment where both B vitamins and cholesterol were absent, but live yeast were present. Unlike vertebrates, which biosynthesize sterols directly from acetate, insects cannot synthesize sterol compounds and must obtain them from their diet or microbial symbionts [58]. Sterols are necessary for proper molting to occur in insects and, thus, are necessary for development and growth. Although the yeast may have helped the brood in the treatments with no cholesterol and B vitamins, development did not differ between ants fed sugar water only and sugar water and yeast. It is unclear why yeast application had little effect in this treatment, although the absence of other micronutrients may have affected the ability of the yeasts to influence ant nutrition. Only exposure to live yeasts in replicates fed the diet minus both B vitamins and cholesterol was associated with the development of more workers. 

Although the heat/chemical defaunation pretreatment may have defaunated the infrabuccal cavity for a limited amount of time in an earlier study [50], we isolated yeasts aside from the test yeast from the buccal cavities at the end of this experiment. It was difficult to keep the experiment completely aseptic and, thus, the ants probably picked up extraneous yeasts from the environment. Several types of yeast were found in the infrabuccal cavities of both non-defaunated and defaunated ants. The presence of these yeasts might be an indication of stress associated with the experimental conditions [36]. 

The heat/chemical defaunation treatment greatly inhibited larval development on all diets compared to larvae developing on the same diets but not exposed to the defaunation pretreatment. The supra-optimal temperature may have stressed the larvae physiologically or it may have killed midgut endosymbiotic bacteria that aid larval nutrition. Pant and Dang [34] used high temperature to defaunate beetle symbionts and found that exposure to 33 °C eliminated their yeast-like symbionts. Fan and Wernegreen [59] found that high-temperature exposure depleted ant bacterial endosymbionts. Cassill and Tschinkel [45] reported that prolonged temperatures did not affect fire ant worker size, but their studies used lower temperatures. They also found that workers exposed to higher temperatures fed larvae less but were more active; thus, larvae still obtained the same amount of food as at lower temperatures. Other work using antibiotic feedings was shown to greatly affect carpenter ant endosymbionts and, subsequently, larval and colony development [14,15,59,60]. These studies exposed ant workers to antibiotic mixtures for longer periods than in our current work, suggesting that our antibiotic/antifungal feeding was not long enough. We do not know why larvae developed so poorly when exposed to the heat and chemical pretreatment in this study. Carpenter ant colonies are frequently found in human habitations, utility poles, and natural tree snags exposed to full sun and high temperatures; however, how they regulate optimal temperature for larval development is not known [61].

The role of B vitamins and sterols supplied by yeasts in the development of eusocial and social hymenoptera has received little attention. The yeast *Saccharomyces cerevisiae* uses the gut tract of the paper wasp *Polistes dominula* as a specialized breeding ground. It is unknown what the yeast supplies to the wasp [31,32]. Stingless bees have been shown to require sterols produced by a yeast associate to develop [33]. *Schwanniomyces polymophus* has been found associated with other ants and has also been shown to reduce complex oligosaccharides to simple sugars [20,62]. Fire ants have been reported to obtain ergosterol from associated yeast species [21]. Research on leaf-cutting ants has shown that ants obtained sterols from their fungal symbiont [29,30]. More recent studies examining these ants suggest that associated yeasts aided in plant biomass conversion of nutrients in their extracellular fungal gardens [27,28]. Early work on carpenter ant nutrition found that ant colonies reared smaller broods on diets without yeast extract, indicating that yeasts or something produced by yeasts was beneficial to brood development [52]. 

In other insects, yeasts have been shown to produce enzymes that aid the insect in digestion of a food source and can produce or convert chemicals that alter insect behavior [63,64,65]. Yeasts associated with desert fruit flies have been shown to aid in the detoxification of compounds on the fruit fly host [63]. Yeasts were found to be responsible for the conversion of aggregation to anti-aggregation pheromones in trees attacked by the beetle *Ips typographus* L. [64]. Yeasts associated with other bark beetles were shown to produce volatiles that inhibited the growth of entomopathogenic fungi [65]. Although not examined in the current study, the potential for yeasts associated with *C. vicinus* to produce volatiles that affect development or colony health might be an interesting follow-up study. These results point to several potential roles for yeasts in carpenter ant development through either direct nutritional contributions or the production of enzymes that facilitate larval digestion processes. The diverse microbial community associated with *C. vicinus* creates a wide array of possible roles for affecting development. These findings with *S. polymorphus* highlight the potential for developing a better understanding of these interrelationships. 

## 5. Conclusions

Exposure of developing *C. vicinus* ant larvae to artificial diets where some components were removed and supplemented with the live yeast *S. polymorphus* was associated with increased brood weight and complete development to the adult stage in cultures where B vitamins and sterols were absent. On all other artificial diets and yeast exposures tested, there were either no effects or deleterious effects of yeast exposure on brood development. The results suggest a supplemental role for this yeast in ant nutrition and development.

## Figures and Tables

**Table 1:** Composition of a complete, holidic, (basal) diet used in nutrient deletion and yeast augmentation tests.

Amino Acids				Water Soluble Vitamins		Fat Soluble Vitamins		Inorganic Salts		Other	
Component	mg/ 100 mL	Component	mg/ 100 mL	Component	mg/ 100 mL	Component	mg/ 100 mL	Component	mg/ 100 mL	Component	mg/ 100 mL
Alanine	100	Leucine	150	Ascorbic acid	10	Cholesterol	50	CaCl_2_	10	Sucrose	10,000
Arginine	100	Lycine	100	Amino benzoic acid	2.5	Linoleic acid	25	CoCl_2_ 6H_2_O	2	Ribonucleic acid	100
Aspartic acid	100	Methionine	50	Biotin	0.2	Linolenic acid	20	CuSO_4_ 5H_2_O	2	KOH	1.5
Cysteine	30	Phenylalanine	100	Calcium Pantothenate	5	Oleic acid	60	FeCl_3_ 6H_2_O	8	K_2_PO_4_	1.5
Glutamic acid	200	Proline	150	Choline Chloride	125	Palmitic acid	60	K_2_PO_4_	50		
Glutamine	50	Serine	100	Folic acid	2.5	Tween 80	500	MgSO_4_ 7H_2_O	60		
Glycine	150	Threonine	75	Inositol	25	Vitamin A	0.1	MnSO_4_ H_2_O	0.5		
Histidine	50	Tryptophan	75	Nicotinic acid	10	Vitamin E	1	NaHPO_4_	5		
Hydroxyproline	30	Tyrosine	100	Pyridoxine	2.5			ZnCl	2		
Isoleucine	100	Valine	100	Riboflavin	0.25						
				Thiamine	2.5						
				Vitamin B12	0.1						

**Table 2:** Average (*n* = 8) brood weights (mg) and standard errors at 2 week intervals over 12 weeks for small worker colonies of *C. vicinus* (non-defaunated) fed various diets chemically defined diets with some nutrients deleted and the same diets augmented with a yeast associate (this table is included because standard error bars are not discernable in Figure 1).

	Week 0		Week 2		Week 4		Week 6		Week 8		Week 10		Week 12	
**Diet/Treatment**	Mean	SE	Mean	SE	Mean	SE	Mean	SE	Mean	SE	Mean	SE	Mean	SE
Basal Diet	0.0055	0.0004	0.0276	0.00032	0.0600	0.0064	0.0755	0.0114	0.0739	0.0100	0.0936	0.0153	0.1015	0.0211
Basal Diet + Yeast	0.0053	0.0005	0.0283	0.0049	0.0584	0.0087	0.0656	0.0111	0.0659	0.0116	0.0896	0.0165	0.0891	0.0171
No B Vitamins	0.0056	0.0004	0.0209	0.0050	0.0403	0.0085	0.0570	0.0088	0.0535	0.0110	0.0701	0.0158	0.0740	0.0163
No B Vitamins + Yeast	0.0051	0.0003	0.0226	0.0041	0.0379	0.0072	0.0493	0.0141	0.0473	0.0116	0.0363	0.0148	0.0419	0.0160
No B Vitamins No Cholesterol	0.0056	0.0003	0.0304	0.0047	0.0535	0.0101	0.0718	0.0129	0.0421	0.0127	0.0191	0.0055	0.0189	0.0060
No B Vitamins No Cholesterol + Yeast	0.0051	0.0008	0.0224	0.0038	0.0511	0.0059	0.0711	0.0087	0.0543	0.0088	0.0559	0.0149	0.0624	0.0188
Sugar Water	0.0050	0.0003	0.0236	0.0024	0.0346	0.0031	0.0326	0.0044	0.0241	0.0035	0.0186	0.0054	0.0219	0.0061
Sugar Water + Yeast	0.0055	0.0005	0.0240	0.0037	0.0279	0.0036	0.0309	0.0029	0.0239	0.0040	0.0245	0.0049	0.0266	0.0069

**Table 3:** Average brood weights and standard errors at 2 week intervals over 12 weeks for small worker colonies of *C. vicinus* defaunated using a two-step heat and chemical defaunation process and then fed chemically defined diets with some nutrients deleted and the same diets augmented with a yeast associate (this is table included because standard error bars are not discernable in Figure 2).

	Week 0		Week 2		Week 4		Week 6		Week 8		Week 10		Week 12	
**Diet/Treatment**	Mean	SE	Mean	SE	Mean	SE	Mean	SE	Mean	SE	Mean	SE	Mean	SE
Heat/Chemical Basal Diet	0.0045	0.0005	0.0186	0.0048	0.0341	0.0078	0.0239	0.0080	0.0218	0.0048	0.0288	0.0082	0.0345	0.0106
Heat/Chemical Basal Diet + Yeast	0.0046	0.0002	0.0148	0.0032	0.0259	0.0041	0.0205	0.0048	0.0215	0.0032	0.0254	0.0041	0.0263	0.0074
Heat/Chemical No B Vitamins	0.0044	0.0004	0.0086	0.0015	0.0111	0.0025	0.0070	0.0016	0.0084	0.0020	0.0143	0.0051	0.0209	0.0091
Heat/Chemical No B Vitamins + Yeast	0.0049	0.0004	0.0088	0.0013	0.0111	0.0020	0.0065	0.0007	0.0090	0.0016	0.0118	0.0029	0.0169	0.0048
Heat/Chemical No B Vitamins No Cholesterol	0.0040	0.0002	0.0089	0.0008	0.0133	0.0023	0.0109	0.0037	0.0108	0.0020	0.0123	0.0034	0.0173	0.0069
Heat/Chemical No B Vitamins No Cholesterol + Yeast	0.0038	0.0003	0.0075	0.0010	0.0120	0.0014	0.0103	0.0028	0.0169	0.0048	0.0223	0.0084	0.0279	0.0109
Heat/Chemical Fed Sugar Water	0.0041	0.0004	0.0164	0.0019	0.0220	0.0028	0.0106	0.0045	0.0118	0.0054	0.0146	0.0063	0.0198	0.0087
Heat/Chemical Fed Sugar Water + Yeast	0.0043	0.0004	0.0153	0.0019	0.0263	0.0031	0.0108	0.0040	0.0111	0.0047	0.0128	0.0044	0.0146	0.0052

## Data Availability

The data presented in this study are available on request from the corresponding author
